# Association of Private Equity Investment in US Nursing Homes With the Quality and Cost of Care for Long-Stay Residents

**DOI:** 10.1001/jamahealthforum.2021.3817

**Published:** 2021-11-19

**Authors:** Robert Tyler Braun, Hye-Young Jung, Lawrence P. Casalino, Zachary Myslinski, Mark Aaron Unruh

**Affiliations:** 1 Division of Health Policy and Economics, Department of Population Health Sciences, Weill Cornell Medical College, New York, New York

## Abstract

**Importance:**

Private equity firms have been acquiring US nursing homes; an estimated 5% of US nursing homes are owned by private equity firms.

**Objective:**

To examine the association of private equity acquisition of nursing homes with the quality and cost of care for long-stay residents.

**Design, Setting, and Participants:**

In this cohort study of 302 private equity nursing homes with 9632 residents and 9562 other for-profit homes with 249 771 residents, a novel national database of private equity nursing home acquisitions was merged with Medicare claims and Minimum Data Set assessments for the period from 2012 to 2018. Changes in outcomes for residents in private equity–acquired nursing homes were compared with changes for residents in other for-profit nursing homes. Analyses were performed from March 25 to June 23, 2021.

**Exposure:**

Private equity acquisitions of 302 nursing homes between 2013 and 2017.

**Main Outcomes and Measures:**

This study used difference-in-differences analysis to examine the association of private equity acquisition of nursing homes with outcomes. Primary outcomes were quarterly measures of emergency department visits and hospitalizations for ambulatory care–sensitive (ACS) conditions and total quarterly Medicare costs. Antipsychotic use, pressure ulcers, and severe pain were examined in secondary analyses.

**Results:**

Of the 259 403 residents in the study (170 687 women [65.8%]; 211 154 White residents [81.4%]; 204 928 residents [79.0%] dually eligible for Medicare and Medicaid; mean [SD] age, 79.3 [5.6] years), 9632 residents were in 302 private equity–acquired nursing homes and 249 771 residents were in 9562 other for-profit homes. The mean quarterly rate of ACS emergency department visits was 14.1% (336 072 of 2 383 491), and the mean quarterly rate of ACS hospitalizations was 17.3% (412 344 of 2 383 491); mean (SD) total quarterly costs were $8050.00 ($9.90). Residents of private equity nursing homes experienced relative increases in ACS emergency department visits of 11.1% (1.7 of 15.3; 1.7 percentage points; 95% CI, 0.3-3.0 percentage points; *P* = .02) and in ACS hospitalizations of 8.7% (1.0 of 11.5; 1.0 percentage point; 95% CI, 0.2-1.1 percentage points; *P* = .003) compared with residents in other for-profit homes; quarterly costs increased 3.9% (270.37 of 6972.04; $270.37; 95% CI, $41.53-$499.20; *P* = .02) or $1081 annually per resident. Private equity acquisition was not significantly associated with antipsychotic use (−0.2 percentage points; 95% CI, −1.7 to 1.4 percentage points; *P* = .83), severe pain (0.2 percentage points; 95% CI, −1.1 to 1.4 percentage points; *P* = .79), or pressure ulcers (0.5 percentage points; 95% CI, −0.4 to 1.3 percentage points; *P* = .30).

**Conclusions and Relevance:**

This cohort study with difference-in-differences analysis found that private equity acquisition of nursing homes was associated with increases in ACS emergency department visits and hospitalizations and higher Medicare costs.

## Key Points

**Question:**

Is private equity acquisition of nursing homes associated with the quality or cost of care for long-stay nursing home residents?

**Findings:**

In this cohort study with difference-in-differences analysis of 9864 US nursing homes, including 9632 residents in 302 nursing homes acquired by private equity firms and 249 771 residents in 9562 other for-profit nursing homes without private equity ownership, private equity acquisition of nursing homes was associated with higher costs and increases in emergency department visits and hospitalizations for ambulatory sensitive conditions.

**Meaning:**

This study suggests that more stringent oversight and reporting on private equity ownership of nursing homes may be warranted.

## Teaser

This cohort study uses a national database of private equity nursing home acquisitions as well as Medicare data to examine the association of private equity acquisition of nursing homes with the quality and cost of care for long-stay residents.

## Introduction

Private equity (PE) investment in US health care has grown dramatically, with $750 billion in deals from 2010 to 2019.^1^ These investments have concerned policy makers because PE firms often create complicated asset, management, and operating structures that may avoid transparency and accountability in patient care. A major target of PE firms has been nursing homes^2^: an estimated 5% of US nursing homes have PE ownership.^3^ For-profit companies are the predominant operators of nursing homes. A large body of research has indicated that for-profit ownership of nursing homes is associated with lower-quality long-term care compared with nonprofit ownership of nursing homes,^4,5^ but little is known about differences in the quality of long-term care provided by PE-owned nursing homes compared with other for-profit nursing homes.

Over half of older adults will eventually stay in a nursing home for postacute or long-term care,^6^ and 12.5% of physicians provide at least some care in these facilities.^7^ There are 1.3 million long-stay nursing home residents in the US, with 90% being 65 years of age or older.^8^ Annual Medicaid expenditures on long-term care total $57 billion^9^ and include the cost of nursing home care for 60% of residents nationally.^8^ High rates of emergency department (ED) visits and hospitalization among residents, which often reflect poor quality long-term care, are associated with a disproportionate share of Medicare spending on this population.^10,11,12^ For these reasons, policy makers have expressed concern about PE acquisitions of nursing homes,^13,14^ concern that has been heightened by the toll of the COVID-19 pandemic on residents of these facilities.^15^

Private equity firm–owned nursing homes may operate differently than other for-profit homes, potentially improving or worsening the quality of care. Private equity firms seek high annual returns of 20% or more.^16^ The pressure to generate high short-term profits could lead to PE firm–owned nursing homes reducing staffing, services, supplies, or equipment, which may have an adverse association with quality of care,^3,17^ whereas non-PE for-profit nursing homes may have business strategies with longer time horizons. Nursing homes purchased by PE firms may be responsible for the debt used by the PE firm as part of a leveraged buyout to acquire the facilities, thereby reducing their financial resources.^18^ Opponents of PE ownership are also concerned that PE firms may not be experienced in nursing home care,^2^ or that they will focus more attention on postacute care and less on long-term care because Medicare reimbursements for patients receiving postacute care are much higher compared with Medicaid payments for long-stay residents.

Conversely, PE acquisitions of nursing homes may lead to higher-quality care through better management and improvements in health information technology capabilities.^19^ Private equity firms may also provide financial and legal resources to improve regulatory compliance, an area in which many nursing homes persistently underperform.^20^

The results of prior studies of PE firm ownership and nursing home quality have been inconsistent.^2,3,21,22,23,24,25,26^ To our knowledge, there have been no national studies of the association between PE firm nursing home ownership and the quality and cost of care for long-stay residents. We used a national sample of long-stay nursing home residents from 2012 through 2018 to compare changes in the quality and cost of care for those in PE firm–acquired nursing homes with residents in for-profit nursing homes without PE investment.

## Methods

### Data Sources

In this cohort study, we identified PE firm nursing home acquisitions using a previously established method (eAppendix in the Supplement).^3^ Acquisitions occurring from 2010 to 2020 were identified using the S&P Capital IQ, Irving Levin Associates Health Care M&A, and Centers for Medicare & Medicaid Services (CMS) Nursing Home Compare Ownership databases, followed by web-based searches. These databases report transactions, including the acquisition announcement date, the name of the acquired nursing home, and the platform nursing home that acquired the facility or the PE firm that owned it. The Nursing Home Compare database provides the CMS Certification Number, nursing home name, address, owner name, and the date that ownership began. This study was approved by the institutional review board of Weill Cornell Medical College, waiving informed consent. The Strengthening the Reporting of Observational Studies in Epidemiology (STROBE) reporting guideline was followed.

We used Medicare fee-for-services claims and Minimum Data Set (MDS) assessments for a nationally representative 20% random sample of beneficiaries from 2012 through 2018. The claims came from the Medicare Provider Analysis and Review File for inpatient stays, the Medicare Carrier File for professional claims, the Medicare Outpatient File for institutional outpatient claims, and the Medicare Hospice File. The Medicare Master Beneficiary Summary File provided information on enrollment, demographics, reason for Medicare entitlement, and chronic and disabling conditions. Minimum Data Set assessments were used to identify long-stay residents (see definition below). These assessments are completed on nursing home admission and at least quarterly thereafter. Medicare claims and MDS assessments were merged using beneficiary identifiers. Measures of nursing home characteristics from Brown University’s LTCFocus^27^ were then merged using nursing home CMS Certification Numbers.

### Study Population

Study participants were long-stay residents, defined as Medicare beneficiaries enrolled in Parts A and B with stays in the same nursing home of 100 days or more, with 10 days or fewer outside the nursing home during this period. Residents aged 65 years or younger were excluded because they have conditions that differ from the broader long-stay population.^28,29,30,31,32^ Similarly, residents of hospital-based nursing homes were excluded (eFigure in the Supplement).^33,34^

### Study Variables

#### Outcome Measures

Outcome measures for the quality and total cost of care were chosen a priori. For our primary analyses, quality outcomes included binary measures for any ambulatory care–sensitive (ACS) ED visit in a given quarter and any ACS hospitalization in a given quarter.^35^ These events should be largely, although not completely, preventable with appropriate care. Our measure of total quarterly costs was constructed by summing Medicare spending on inpatient, outpatient, postacute, hospice, and professional services in addition to laboratory tests. Although Medicaid covers long-term care in nursing homes for many residents, these costs are generally fixed daily payments, and poorer quality long-term care is likely reflected in increased spending for Medicare-covered services rather than in Medicaid spending because Medicare pays for ED visits and hospitalizations.^12^

In secondary analyses, we examined additional quality outcomes, including dichotomous measures for any antipsychotic medication use, presence of a pressure ulcer, and self-reported severe pain (values >6 on a 10-point pain scale, where 0 indicates no pain and 10 indicates the worst pain). We examined these outcomes owing to their relevance for long-stay residents, as indicated in the literature,^36,37,38,39^ but include them in secondary analyses because they are self-reported by nursing homes and may therefore be biased.

#### Resident and Nursing Home Characteristics

Resident characteristics included age, race and ethnicity, sex, dual eligibility for Medicare and Medicaid, indicators for 66 chronic and potentially disabling conditions used for risk adjustment (eTable 2 in the Supplement), activities of daily living score at initial assessment (range, 1-28, where a higher score indicates a greater need for assistance with activities of daily living), and severe cognitive impairment (score >3 on the 4-point Cognitive Function Scale, where 1 indicates mild impairment and 4 indicates severe impairment).^40^ Information on race and ethnicity in Medicare claims is generally collected from the Social Security Administration. We included measures for White and Black individuals. A third category for all other race and ethnicity categories (Asian, Hispanic, North American Native, and other) in Medicare claims was also included; we did not use separate measures for these groups because they are not accurately identified in Medicare claims.^41^ Nursing home characteristics included occupancy rate, multifacility chain affiliation, total number of beds, and terciles of the distributions of the percentage of patients covered by Medicare and the percentage covered by Medicaid.

### Statistical Analysis

Statistical analyses were conducted using Stata MP, version 16.1 (StataCorp LLC). Analyses were performed from March 25 to June 23, 2021. Our treatment group included residents of nursing homes acquired by PE firms between 2013 and 2017. The control group included residents of for-profit nursing homes without PE ownership and located in a Hospital Referral Region with at least 1 PE firm–owned nursing home. We excluded nursing homes acquired by PE firms prior to 2013 and after 2017 so that all facilities in the treatment group had at least 1 year of data before (2012) and after acquisition (2018). If a nursing home was reacquired by a PE firm during our study period, we considered only the first acquisition in the treatment group. Nursing homes reacquired in 2013 or later after being acquired prior to 2013 were excluded.

We conducted 4 analyses. First, we examined the prevalence and geographical distribution of PE firm nursing home acquisitions between 2013 and 2017. Second, we compared resident and facility characteristics of PE and non-PE facilities in 2012 (before acquisition) to assess whether acquired nursing homes were different than nonacquired homes. Third, we compared the resident and facility characteristics of preacquisition nursing homes with postacquisition nursing homes using *t* tests for continuous variables and proportion tests for categorical variables. Fourth, we used a difference-in-differences approach to examine changes in outcomes associated with PE firm acquisition. Our variable of interest was an interaction between an indicator identifying PE firm–acquired nursing homes and an indicator for the postacquisition period; the corresponding estimate represents the association of PE acquisition with the outcome. Other covariates included resident and nursing home characteristics in addition to fixed effects for quarter, year, nursing home, Hospital Referral Region, and Hospital Referral Region interaction with year.

We used linear regression models for all outcomes, with SEs clustered at the nursing home level. The unit of analysis was the resident quarter for ACS ED visits, ACS hospitalizations, and total costs. In the secondary analyses, antipsychotic use, presence of a pressure ulcer, and severe pain occurring between nursing home admission and the first quarterly MDS assessment were examined. For unadjusted and adjusted results, relative differences were calculated by dividing each estimate by the unadjusted mean of the outcome in the preacquisition period.

We conducted 3 sensitivity analyses. The first addressed potential measurement error in the acquisition date. In some cases, we captured the date on which an acquisition was publicly announced, which may not be the exact date a nursing home was acquired. We addressed this issue by excluding the calendar year of the announced acquisition as a washout period. The second analysis included only nursing homes present in all years of the study period to mitigate the possibility of results being associated with facilities entering and leaving the sample. The third analysis compared differences in preacquisition outcome trends between residents of PE firm–owned and non-PE firm–owned nursing homes to test the parallel trends assumption for our difference-in-differences approach. All *P* values were from 2-sided tests and results were deemed statistically significant at *P* < .05.

## Results

### PE Investment in Nursing Homes

The Figure presents the geographical distribution of PE firm nursing home acquisitions between 2013 and 2017. We identified 79 PE transactions (eTable 1 in the Supplement), representing 302 nursing homes and 37 states, concentrated mostly in California, Kentucky, Massachusetts, Ohio, Tennessee, Texas, and Washington.

**Figure.**
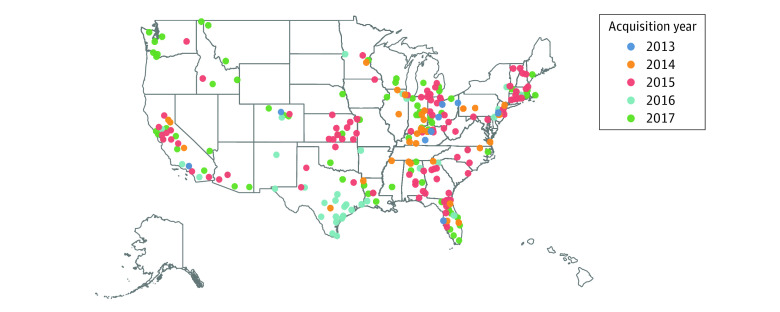
Locations of Nursing Homes Acquired by Private Equity Firms, 2013-2017 There were 6 transactions representing 9 nursing homes in 2013, 15 transactions representing 34 nursing homes in 2014, 30 transactions representing 114 nursing homes in 2015, 12 transactions representing 43 nursing homes in 2016, and 16 transactions representing 102 nursing homes in 2017.

### Characteristics of Residents and Nursing Homes

Table 1 presents resident and nursing home characteristics for the entire sample (pooled sample, 2012-2018) and for PE firm–owned and non-PE firm–owned for-profit nursing homes both before acquisition (2012) and after acquisition (2018). Of the 259 403 residents in the study, 170 687 (65.8%) were women, 211 154 (81.4%) were White, and 204 928 (79.0%) were dually eligible for Medicare and Medicaid; the mean (SD) age was 79.3 (5.6) years. In 2012, there were 289 nursing homes that were later acquired by PE firms and 7954 for-profit homes that were never acquired. In 2018, there were 295 PE firm–owned nursing homes and 8323 for-profit homes that were never acquired by PE firms. During the study period (2012-2018), there was a total of 9632 long-stay residents in 302 PE firm–owned nursing homes and 249 771 residents in 9562 non-PE firm–owned nursing homes.

**Table 1.** Characteristics of Residents and Nursing Homes

Characteristic	2012-2018 Pooled sample (n = 259 403)^a^	Preacquisition period (2012)				Postacquisition period (2018)		
		All (n = 65 670)^b^	PE (n = 2209)	For-profit (n = 63 461)	Unadjusted difference	PE (n = 995)	For-profit (n = 27 804)	Unadjusted difference
Nursing homes, No.	9864	8243	289	7954	NA	295	8323	NA
Nursing home characteristics								
Occupancy rate, %^c^	82.7	83.7	83.5	83.7	0.2	82.5	81.2	1.5
Chain facility, No. (%)	5889 (59.7)	4797 (58.2)	220 (76.1)	4567 (57.4)	18.6	254 (86.1)	5094 (61.2)	6.4
Total beds, mean (SD), No.	130.5 (63.7)	132.5 (62.8)	135.4 (69.5)	132.4 (62.6)	3.0	120.1 (51.8)	129.0 (64.6)	−11.9
Medicare residents by tercile, %^c^								
Lowest	31.2	29.7	20.6	30.0	−9.4	26.5	37.0	−1.1
Middle	33.8	35.7	37.6	36.0	1.6	33.4	31.7	0.1
Highest	35.0	34.6	41.2	34.3	7.5	40.1	31.4	1.2
Medicaid residents by tercile, %^c^								
Lowest	27.0	22.1	25.6	22.0	3.6	25.4	29.4	−7.6
Middle	35.2	35.3	38.5	35.2	3.3	36.7	33.2	0.2
Highest	37.8	42.6	35.9	42.8	−6.9	37.9	37.5	7.3
Resident characteristics								
Age group, No. (%), y								
65-69	48 768 (18.7)	14 119 (21.5)	495 (22.4)	13 644 (21.5)	0.9	169 (17.0)	166 (16.7)	−0.06
70-74	25 681 (9.9)	6567 (10.0)	221 (10.0)	6346 (10.0)	0	131 (13.2)	109 (11.0)	2.2
75-79	32 944 (12.7)	8471 (12.9)	305 (13.8)	8123 (12.8)	1.0	143 (14.4)	134 (13.5)	−0.1
80-84	43 580 (16.8)	11 098 (16.9)	398 (18.0)	10 788 (17.0)	1.0	164 (16.5)	167 (16.8)	−1.3
≥85	108 690 (41.9)	25 414 (38.7)	800 (36.2)	24 750 (39.0)	−2.8	397 (39.9)	421 (42.3)	−0.5
Race, No. (%)								
Black	34 501 (13.0)	10 179 (15.5)	274 (12.4)	9963 (15.7)	−3.3	87 (8.7)	122 (12.3)	−0.3
White	211 154 (81.4)	51 789 (79.0)	1754 (79.4)	50 134 (79.0)	0.4	837 (84.1)	818 (82.2)	1.5
Other non-White^d^	13 748 (5.3)	3481 (5.3)	175 (7.9)	3300 (5.2)	2.7	66 (6.6)	51 (5.1)	−1.3
Female, No. (%)	170 687 (65.8)	44 459 (67.7)	1445 (65.4)	43 027 (67.8)	−2.4	621 (62.4)	638 (64.1)	0.7
Dual eligibility for Medicare and Medicaid, No. (%)	204 928 (79.0)	10 310 (85.5)	1871 (84.7)	54 259 (85.5)	−0.8	776 (78.0)	776 (78.0)	0.8
Baseline ADL score, mean (SD) (range, 1-28)	15.9 (6.7)	15.7 (7.3)	16.3 (7.2)	15.7 (7.3)	0.6	16.4 (5.7)	16.0 (6.2)	−0.3
Severe cognitive impairment, No. (%)	259 (0.1)	131 (0.2)	7 (0.3)	127 (0.2)	0.1	1 (0.1)	1 (0.1)	−0.1

Abbreviations: ADL, activities of daily living; NA, not applicable; PE, private equity.

^a^ The pooled sample consists of all resident observations from 2012 to 2018.

^b^ The complete sample consists of all resident observations in 2012.

^c^ This is a facility-level measure; numerator and denominator data are not available.

^d^ Other non-White is defined as a category for all other race and ethnicity categories in Medicare claims (Asian, Hispanic, North American Native, and other).

In the pooled sample, nursing homes had a mean (SD) of 130.5 (63.7) beds and a mean (SD) occupancy rate of 82.7% (12.3%); 59.7% of facilities (5889 of 9864) were part of a chain (Table 1). Residents had a mean (SD) baseline activities of daily living score of 15.9 (6.7); 259 residents (0.1%) had severe cognitive impairment, and 108 690 (41.9%) were 85 years of age or older. Prior to acquisition, 75.8% of PE firm–owned nursing homes (219 of 289) and 57.4% of non-PE firm–owned nursing homes (4566 of 7954) were part of a chain. Private equity firm–owned nursing homes had a mean (SD) total bed count of 135.4 (69.5), and non-PE firm–owned nursing homes had a mean total bed count of 132.4 (62.6). A larger percentage of PE nursing homes was in the highest tercile of the percentage of residents covered by Medicare (41.2% vs 34.3%). Resident mean (SD) baseline activities of daily living scores for PE firm–owned and non-PE firm–owned nursing homes were 16.3 (7.2) and 15.7 (7.3), respectively. The mean percentage of Black residents, female residents, and residents aged 85 years or older were 12.4% (274 of 2209), 65.4% (1445 of 2209), and 36.2% (800 of 2209), respectively, for PE firm–owned nursing homes and 15.7% (9963 of 63 461), 67.8% (43 027 of 63 461), and 39.0% (24 750 of 63 461), respectively, for non-PE firm–owned nursing homes.

After acquisition, PE firm–owned nursing homes had a 1.5% higher mean occupancy rate and 6.4% more facilities affiliated with chains compared with non-PE firm–owned homes (Table 1). The mean number of beds of PE firm–owned nursing homes was 11.9 beds lower after acquisition. Following acquisition, PE firm–owned nursing homes had a 7.6% decrease in the lowest tercile for the percentage of residents covered by Medicaid and a 7.3% increase in the highest tercile for the percentage of residents covered by Medicaid.

### Changes in Outcomes After Acquisition

Table 2 presents unadjusted and adjusted difference-in-differences results. Among all residents, mean quarterly rates of ACS ED visits and ACS hospitalizations, respectively, were 14.1% (336 072 of 2 383 491) and 17.3% (412 344 of 2 383 491); mean (SD) total quarterly Medicare costs were $8050.00 ($9.90). Preacquisition, there were no statistically significant differences in unadjusted outcomes. In the unadjusted difference-in-differences results, PE firm–owned nursing homes had a 13.1% relative increase in ACS ED visits (2.0 of 15.3; 2.0 percentage points; 95% CI, 1.0-4.0 percentage points; *P* = .01) and a 10.4% relative increase in ACS hospitalizations (1.2 of 11.5; 1.2 percentage point; 95% CI, 0.01-2.3; *P* = .04) compared with changes during the same time period in non-PE firm–owned nursing homes. There was no statistically significant difference in the unadjusted comparison of total costs ($94.02; 95% CI, −$392.42 to $580.50; *P* = .85).

**Table 2.** Changes in Quality and Costs for Long-Stay Nursing Home Residents After PE Firm Acquisition Compared With For-Profit Nursing Homes Without PE Firm Ownership^a^

Outcome	Pooled sample, 2012-2018, No. (%)^b^	Preacquisition period, 2012				Postacquisition period, 2018			Differential change				Relative change, %^c^
		All	PE	For-profit	Unadjusted difference	PE	Non-PE	Unadjusted difference	Unadjusted (95% CI)	*P* value	Adjusted (95% CI)	*P* value	
Quality measures													
Emergency department visit (n = 2 383 491)	336 072 (14.1)	15.3	15.3	15.3	0	20.1	18.1	2.0	2.0 (1.0 to 4.0)	.01	1.7 (0.3 to 3.0)	.02	11.1
Hospitalization (n = 2 383 491)	412 344 (17.3)	11.5	10.4	11.5	−1.1	14.6	14.5	0.1	1.2 (0.01 to 2.3)	.04	1.0 (0.2 to 1.1)	.003	8.7
Cost measure													
Total costs (n = 2 383 491), mean (SD), $	8050.00 (9.90)	6972.04 (39.60)	7066.26 (208.72)	6968.43 (40.30)	97.83 (212.60)	8818.60 (126.30)	8626.75 (24.84)	191.85 (28.72)	94.02 (−392.42 to 580.50)	.85	270.37 (41.53 to 499.20)	.02	3.9

Abbreviation: PE, private equity.

^a^ Linear regressions were used for estimation. All models included the following covariates: age group (65-69, 70-74, 75-79, 80-84, and ≥85 years), race and ethnicity (Black, White, other non-White race [Asian, Hispanic, North American Native, and other]), sex, dual eligibility for Medicare and Medicaid, indicators for 66 chronic and disabling conditions used for risk adjustment (see eTable 2 in the Supplement for a list of the chronic conditions), activities of daily living score at initial assessment (range, 1-28, where a higher score indicates a greater need for assistance with activities of daily living)), and severe cognitive impairment (scores >3 on the 4-point Cognitive Function Scale). Nursing home characteristics included occupancy rate, an indicator for multifacility affiliation, total number of beds, and terciles of the distributions of the percentage of patients covered by Medicare and the percentage covered by Medicaid. Other covariates included fixed effects for quarter, year, nursing home, Hospital Referral Region, and Hospital Referral Region interaction with year. The unit of analysis is at the resident-quarter level. Standard errors were adjusted for clustering at the level of the nursing home.

^b^ The pooled sample consists of all resident observations from 2012 to 2018.

^c^ Relative changes were derived from the sample by dividing the adjusted estimates for all outcomes by the unadjusted mean of the outcomes in the preacquisition period (2012).

In adjusted differences-in-differences comparisons, PE firm acquisition was associated with an 11.1% relative increase in ACS ED visits (1.7 of 15.3; 1.7 percentage points; 95% CI, 0.3-3.0 percentage points; *P* = .02), an 8.7% relative increase in ACS hospitalizations (1.0 of 11.5; 1.0 percentage point; 95% CI, 0.2-1.1 percentage points; *P* = .003), and a 3.9% relative increase in total quarterly costs ($270.37 of $6972.04; $270.37; 95% CI, $41.53-$499.20; *P* = .02) (Table 2).

In the secondary analyses, there were no statistically significant adjusted estimates associated with PE firm acquisition of nursing homes in the use of antipsychotics (−0.2 percentage points; 95% CI, −1.7 to 1.4 percentage points; *P* = .83), pressure ulcers (0.5 percentage points; 95% CI, −0.4 to 1.3; *P* = .30), or severe pain (0.2 percentage points; 95% CI, −1.1 to 1.4 percentage points; *P* = .79) (Table 3).

**Table 3.** Changes in Quality Measures for Long-Stay Nursing Home Residents After PE Firm Acquisition Compared With For-Profit Nursing Homes Without PE Firm Ownership Examined in Secondary Analyses^a^

Minimum data set quality measure	Pooled sample, 2012-2018, No. (%)^b^	Preacquisition period (2012)				Postacquisition period (2018)			Differential change				Relative change, %^c^
		All	PE	For-profit	Unadjusted difference	PE	Non-PE	Unadjusted difference	Unadjusted (95% CI)	*P* value	Adjusted (95% CI)	*P* value	
Antipsychotic medications (n = 230 687)	49 598 (21.5)	22.1	20.8	22.2	−1.4	16.8	19.3	−2.5	−1.1 (−4.8 to 2.4)	.53	−0.2 (−1.7 to 1.4)	.83	−0.9
Pressure ulcer (n = 278 188)	13 631 (4.9)	3.6	3.8	3.6	0.2	5.0	5.6	−0.6	0.8 (−2.4 to 0.7)	.26	0.5 (−0.4 to 1.3)	.30	13.5
Severe pain (n = 217 284)	16 731 (7.7)	9.4	9.1	9.4	−0.3	4.9	5.3	−0.4	−0.1 (−2.5 to 2.3)	.94	0.2 (−1.1 to 1.4)	.79	−0.2

Abbreviation: PE, private equity.

^a^ Linear regressions were used for estimation. All models included the following covariates: age group (65-69, 70-74, 75-79, 80-84, and ≥85 years), race and ethnicity (Black, White, and other non-White race [Asian, Hispanic, North American Native, and other]), sex, dual eligibility for Medicare and Medicaid, indicators for 66 chronic and disabling conditions used for risk adjustment (see eTable 2 in the Supplement for a list of the chronic conditions), activities of daily living score at initial assessment (range, 1-28, where a higher score indicates a greater need for assistance with activities of daily living), and severe cognitive impairment (scores >3 on the 4-point Cognitive Function Scale). Nursing home characteristics included occupancy rate, an indicator for multifacility chain affiliation, total number of beds, and terciles of the distributions of the percentage of patients covered by Medicare and the percentage covered by Medicaid. Other covariates included fixed effects for quarter, year, nursing home, Hospital Referral Region, and Hospital Referral Region interaction with year. Standard errors were adjusted for clustering at the level of the nursing home.

^b^ The pooled sample consists of all resident observations from 2012 to 2018.

^c^ Relative changes were derived from the sample by dividing the adjusted estimates for all outcomes by the unadjusted mean of the outcomes in the preacquisition period (2012).

### Sensitivity Analyses

When a washout period for the year of PE firm acquisition was used, the results remained mainly consistent with our primary analyses, although the estimate for total costs was no longer statistically significant (eTable 3 in the Supplement). After limiting the sample to nursing homes present in all years of the study period (275 PE firm–owned nursing homes and 8407 for-profit, non-PE firm–owned homes), the results remained consistent with the primary analyses (eTable 4 in the Supplement). Tests to assess differences in preacquisition trends did not show any meaningful differences (eTable 5 in the Supplement).

## Discussion

In this national cohort study with adjusted difference-in-differences analysis, long-stay residents of PE firm–owned nursing homes were 11.1% more likely to have an ACS ED visit and 8.7% more likely to experience an ACS hospitalization after acquisition compared with residents of non-PE firm–owned, for-profit nursing homes and had total Medicare costs that were 3.9% higher (approximately $1080 annually per resident). There were no differences between PE firm–owned nursing homes and non-PE firm–owned nursing homes in the likelihood of residents receiving antipsychotics, developing a pressure ulcer, or experiencing severe pain.

Prior studies of PE firm ownership and nursing home quality have had mixed results. Two studies that included samples of nursing homes from more than 1 decade ago did not find an association between PE firm ownership and quality based on MDS measures and the results of state inspections, but they did not assess ACS ED visits or hospitalizations or costs.^2,24^ One of the studies did not distinguish between postacute and long-stay residents,^2^ and the other study was limited to long-stay residents in Ohio.^24^ Three studies found little evidence of an association between PE firm ownership and staffing,^2,21,22^ while another study found reduced staffing at PE firm–owned nursing homes.^23^ One recent working paper found increased registered nurse staffing and improved five-star ratings in PE firm–owned facilities in more competitive markets compared with non-PE firm–owned nursing homes.^26^ A second working paper found PE firm ownership to be associated with increased mortality rates and higher costs for postacute patients, declines in five-star ratings, and slightly lower levels of direct care staffing, with the exception of an increase in registered nurse staffing.^25^ A recent study during the COVID-19 pandemic found that PE firm–owned facilities performed similarly to those with other types of ownership in the number of COVID-19 cases and deaths, but PE firm–owned nursing homes had lower supplies of personal protective equipment.^3^

Public funds from the Medicare and Medicaid programs are the largest sources of nursing home revenue, but lack of transparency in ownership makes it very difficult to identify PE firm acquisitions of nursing homes and difficult to compare types of homes. The CMS requires that ownership stakes of 5% or more be reported in the Provider Enrollment, Chain, and Ownership System (PECOS). However, PECOS is not publicly available, even generally speaking, to researchers, and the information reported in PECOS is not regularly audited. Private equity firms frequently use complex corporate structures that make it difficult to identify related third parties.^42^ Tracking the amount of revenue for staffing, services, and supplies that goes to multiple related or co-owned entities that appear in PECOS as having ownership stakes in a nursing home is often not possible. Our findings suggest that more stringent oversight and reporting of related entities may be warranted. Policy makers might consider making more detailed ownership information available in outlets that provide consumers with information on nursing home quality, such as Nursing Home Compare.

### Limitations

Our study has some limitations. First, we identified PE firm nursing home ownership using the S&P Capital IQ and Irving Levin Associates Health Care M&A databases, which rely on public announcements of acquisitions and may not include smaller acquisitions. Second, the CMS Nursing Home Ownership file may not always identify the parent company of a platform nursing home. In this case, nursing homes acquired on behalf of a PE firm by these platform nursing homes may appear to be for-profit nursing homes without PE firm ownership; this factor would bias our results toward the null. Third, we were unable to track PE firm exits from nursing homes, which also would bias our estimates toward no association of ownership with outcomes. However, PE firms usually sell acquired organizations within 3 to 7 years, so there were likely few exits during the 2013-2017 period used for our analyses.^43^ Fourth, our study population included only residents participating in Medicare fee-for-service. As of 2018, 39% of all Medicare beneficiaries participated in Medicare Advantage,^44^ although the participation rate of nursing home patients is estimated to be just over half the rate of the broader Medicare population.^45^ Medicare Advantage claims are incomplete and therefore not ideal for measuring quality or costs.^46^ Fifth, we did not compare PE firm–owned nursing homes with nonprofit nursing homes. However, previous research indicates that nonprofit nursing homes have better quality than for-profit nursing homes.^4,5^

## Conclusions

This cohort study suggests that PE firm–owned nursing homes provided somewhat lower-quality long-term care than other for-profit homes based on 2 widely used quality measures and were associated with higher total per-beneficiary Medicare costs.

## Notes

Supplement. **eAppendix.** Identification of Private Equity Nursing Homes **eFigure.** Sample Selection **eTable 1.** Total Acquisitions by Private Equity Firms **eTable 2.** Chronic and Potentially Disabling Conditions **eTable 3.** Changes in Quality and Costs After Private Equity Acquisition Compared With For-Profit Nursing Homes Without Private Equity Ownership Using a Washout Period **eTable 4.** Changes in Quality and Costs After Private Equity Acquisition Compared With For-Profit Nursing Homes Without Private Equity Ownership Using a Balanced Nursing Home Panel **eTable 5.** Pre-Trend Tests to Examine Outcomes of Private Equity Nursing Homes and For-Profit Nursing Homes Without Private Equity Ownership Prior to Acquisition

## References

[1] Scheffler RM, Alexander LM, Godwin JR. Soaring private equity investment in the healthcare sector: consolidation accelerated, competition undermined, and patients at risk. The Nicholas C. Petris Center on Health Care Markets and Consumer Welfare. Published May 19, 2021. Accessed September 27, 2021. https://petris.org/soaring-private-equity-investment-in-the-healthcare-sector-consolidation-accelerated-competition-undermined-and-patients-at-risk/

[2] Stevenson DG, Grabowski DC. Private equity investment and nursing home care: is it a big deal? Health Aff (Millwood). 2008;27(5):1399-1408. doi:10.1377/hlthaff.27.5.1399 18780930

[3] Braun RT, Yun H, Casalino LP, . Comparative performance of private equity–owned US nursing homes during the COVID-19 pandemic. JAMA Netw Open. 2020;3(10):e2026702. doi:10.1001/jamanetworkopen.2020.26702 33112402 PMC7593807

[4] Comondore VR, Devereaux PJ, Zhou Q, . Quality of care in for-profit and not-for-profit nursing homes: systematic review and meta-analysis. BMJ. 2009;339:b2732. doi:10.1136/bmj.b2732 19654184 PMC2721035

[5] Hirth RA, Grabowski DC, Feng Z, Rahman M, Mor V. Effect of nursing home ownership on hospitalization of long-stay residents: an instrumental variables approach. Int J Health Care Finance Econ. 2014;14(1):1-18. doi:10.1007/s10754-013-9136-3 24234287 PMC3969758

[6] Hurd MD, Michaud PC, Rohwedder S. Distribution of lifetime nursing home use and of out-of-pocket spending. Proc Natl Acad Sci U S A. 2017;114(37):9838-9842. doi:10.1073/pnas.1700618114 28847934 PMC5603996

[7] Jung HY, Qian Y, Katz PR, Casalino LP. The characteristics of physicians who primarily practice in nursing homes. J Am Med Dir Assoc. 2021;22(2):468-469. doi:10.1016/j.jamda.2020.10.006 33234449 PMC9288658

[8] Harris-Kojetin L, Sengupta M, Park-Lee E, . Long-term care providers and services users in the United States: data from the National Study of Long-Term Care Providers, 2013-2014. Vital Health Stat 3. 2016(38):x-xii; 1-105. 27023287

[9] Medicaid and CHIP Payment and Access Commission. Long-term services and supports: nursing faciilities. Accessed April 29, 2021. https://www.macpac.gov/subtopic/nursing-facilities/

[10] McCarthy EP, Ogarek JA, Loomer L, . Hospital transfer rates among US nursing home residents with advanced illness before and after initiatives to reduce hospitalizations. JAMA Intern Med. 2020;180(3):385-394. doi:10.1001/jamainternmed.2019.6130 31886827 PMC6990757

[11] Grabowski DC, Stevenson DG, Caudry DJ, . The impact of nursing home pay-for-performance on quality and Medicare spending: results from the nursing home value-based purchasing demonstration. Health Serv Res. 2017;52(4):1387-1408. doi:10.1111/1475-6773.12538 27491950 PMC5517679

[12] Unruh MA, Grabowski DC, Trivedi AN, Mor V. Medicaid bed-hold policies and hospitalization of long-stay nursing home residents. Health Serv Res. 2013;48(5):1617-1633. doi:10.1111/1475-6773.12054 23521571 PMC3737418

[13] Senators Warren, Brown, and Rep Pocan investigate the role of private equity in declining quality of nursing home care. Accessed June 30, 2020. Elizabeth Warren. https://www.warren.senate.gov/oversight/letters/senators-warren-brown-and-rep-pocan-investigate-role-of-private-equity-in-declining-quality-of-nursing-home-care

[14] Ways & Means Committee. Pascrell opening statement at oversight subcommittee hearing on examining private equity’s role in the U.S. health care system. Updated March 25, 2021. Accessed May 26, 2021. https://waysandmeans.house.gov/media-center/press-releases/pascrell-opening-statement-oversight-subcommittee-hearing-examining

[15] Hirsch L. Elizabeth Warren demands answers from private equity industry on coronavirus response, lobbying. CNBC. June 25, 2020. Accessed September 30, 2021. https://www.cnbc.com/2020/06/25/elizabeth-warren-demands-answers-from-private-equity-on-coronavirus-lobbying.html

[16] Casalino LP, Saiani R, Bhidya S, Khullar D, O’Donnell E. Private equity acquisition of physician practices. Ann Intern Med. 2019;171(1):78. doi:10.7326/L19-0256 31261393

[17] Kaiser Health News. Congressional panels examine effects of private equity ownership of nursing homes. June 11, 2009. Accessed July 12, 2020. https://khn.org/morning-breakout/dr00048912/

[18] Pradhan R, Weech-Maldonado R, Harman JS, Laberge A, Hyer K. Private equity ownership and nursing home financial performance. Health Care Manage Rev. 2013;38(3):224-233. doi:10.1097/HMR.0b013e31825729ab 22609748

[19] Vest JR, Jung HY, Wiley K Jr, Kooreman H, Pettit L, Unruh MA. Adoption of health information technology among US nursing facilities. J Am Med Dir Assoc. 2019;20(8):995-1000.e4. doi:10.1016/j.jamda.2018.11.002 30579920 PMC6591108

[20] Harrington C, Carrillo H, Garfield R, Squires E. Nursing facilities, staffing, residents and facility deficiencies, 2009 through 2016. Kaiser Family Foundation. April 3, 2018. Accessed September 30, 2020. https://www.kff.org/medicaid/report/nursing-facilities-staffing-residents-and-facility-deficiencies-2009-through-2016/

[21] Harrington C, Olney B, Carrillo H, Kang T. Nurse staffing and deficiencies in the largest for-profit nursing home chains and chains owned by private equity companies. Health Serv Res. 2012;47(1, pt 1):106-128. doi:10.1111/j.1475-6773.2011.01311.x 22091627 PMC3447240

[22] Bos A, Harrington C. What happens to a nursing home chain when private equity takes over? a longitudinal case study. Inquiry. 2017;54:1-10. doi:10.1177/0046958017742761 29161948 PMC5798733

[23] Pradhan R, Weech-Maldonado R, Harman JS, Hyer K. Private equity ownership of nursing homes: implications for quality. J Health Care Finance. 2014;42(2):1-14.

[24] Huang SS, Bowblis JR. Private equity ownership and nursing home quality: an instrumental variables approach. Int J Health Econ Manag. 2019;19(3-4):273-299. doi:10.1007/s10754-018-9254-z 30357589

[25] Gupta A, Howell ST, Yannelis C, Gupta A. Does Private Equity Investment in Healthcare Benefit Patients? Evidence From Nursing Homes. Working paper 28474. NBER working paper series. National Bureau of Economic Research; 2021.

[26] Gandhi A, Song Y, Upadrashta P. Private equity, consumers, and competition: evidence from the nursing home industry. June 19, 2020. Accessed May 26, 2021. https://papers.ssrn.com/sol3/papers.cfm?abstract_id=3626558

[27] LTCfocus. Brown School of Public Health. Who We Are. Accessed May 14, 2021. http://www.ltcfocus.org

[28] Aschbrenner K, Grabowski DC, Cai S, Bartels SJ, Mor V. Nursing home admissions and long-stay conversions among persons with and without serious mental illness. J Aging Soc Policy. 2011;23(3):286-304. doi:10.1080/08959420.2011.579511 21740203 PMC3815475

[29] Buchanan RJ, Wang S, Huang C, Graber D. Profiles of nursing home residents with multiple sclerosis using the minimum data set. Mult Scler. 2001;7(3):189-200. doi:10.1177/135245850100700310 11475444

[30] Buchanan RJ, Wang S, Huang C. Profiles of nursing home residents with HIV. J Health Care Poor Underserved. 2002;13(3):379-391. doi:10.1353/hpu.2010.0698 12152507

[31] Buchanan RJ, Gorman DM, Wang S, Huang C. Co-morbidity and treatment needs among nursing home residents receiving alcohol and drug treatment. J Addict Dis. 2003;22(2):31-47. doi:10.1300/J069v22n02_03 12703667

[32] Lair TJ. A Profile of 1987 Nursing Home Users Under 65: National Medical Expenditure Survey Research Findings 13. Agency for Health Care Policy and Research; 1992.

[33] Ouslander JG, Lamb G, Tappen R, . Interventions to reduce hospitalizations from nursing homes: evaluation of the INTERACT II collaborative quality improvement project. J Am Geriatr Soc. 2011;59(4):745-753. doi:10.1111/j.1532-5415.2011.03333.x 21410447

[34] Rahman M, Norton EC, Grabowski DC. Do hospital-owned skilled nursing facilities provide better post-acute care quality? J Health Econ. 2016;50:36-46. doi:10.1016/j.jhealeco.2016.08.004 27661738 PMC5127756

[35] Agency for Healthcare Research and Quality. Prevention quality indicators, 2017. Accessed January 29, 2021. https://www.qualityindicators.ahrq.gov/Modules/all_resources.aspx

[36] Jung HY, Meucci M, Unruh MA, Mor V, Dosa D. Antipsychotic use in nursing home residents admitted with hip fracture. J Am Geriatr Soc. 2013;61(1):101-106. doi:10.1111/jgs.12043 23252409 PMC3854869

[37] Maust DT, Kim HM, Chiang C, Kales HC. Association of the Centers for Medicare & Medicaid Services’ national partnership to improve dementia care with the use of antipsychotics and other psychotropics in long-term care in the United States from 2009 to 2014. JAMA Intern Med. 2018;178(5):640-647. doi:10.1001/jamainternmed.2018.0379 29550856 PMC5885206

[38] McGarry BE, Joyce NR, McGuire TG, Mitchell SL, Bartels SJ, Grabowski DC. Association between high proportions of seriously mentally ill nursing home residents and the quality of resident care. J Am Geriatr Soc. 2019;67(11):2346-2352. doi:10.1111/jgs.16080 31355443 PMC6861617

[39] Wei YJJ, Solberg L, Chen C, . Pain assessments in MDS 3.0: agreement with vital sign pain records of nursing home residents. J Am Geriatr Soc. 2019;67(11):2421-2422. doi:10.1111/jgs.16122 31403708 PMC6861605

[40] Thomas KS, Dosa D, Wysocki A, Mor V. The Minimum Data Set 3.0 Cognitive Function Scale. Med Care. 2017;55(9):e68-e72. doi:10.1097/MLR.0000000000000334 25763665 PMC4567556

[41] Filice CE, Joynt KE. Examining race and ethnicity information in Medicare administrative data. Med Care. 2017;55(12):e170-e176. doi:10.1097/MLR.0000000000000608 29135782

[42] Harrington C, Montgomery A, King T, Grabowski DC, Wasserman M. These administrative actions would improve nursing home ownership and financial transparency in the post COVID-19 period. Health Affairs blog. February 11, 2021. Accessed May 27, 2021. https://www.healthaffairs.org/do/10.1377/hblog20210208.597573/full/

[43] Braun RT, Bond AM, Qian Y, Zhang M, Casalino LP. Private equity in dermatology: effect on price, utilization, and spending. Health Aff (Millwood). 2021;40(5):727-735. doi:10.1377/hlthaff.2020.02062 33939519

[44] Koma W, Cubanski J, Neuman T. A snapshot of sources of coverage among Medicare beneficiaries in 2018. Kaiser Family Foundation. March 23, 2021. Accessed September 25, 2021. https://www.kff.org/medicare/issue-brief/a-snapshot-of-sources-of-coverage-among-medicare-beneficiaries-in-2018/

[45] Jung HY, Li Q, Rahman M, Mor V. Medicare Advantage enrollees’ use of nursing homes: trends and nursing home characteristics. Am J Manag Care. 2018;24(8):e249-e256. 30130025 PMC6225776

[46] Medicare Payment Advisory Commission. Report to Congress: Medicare and the health care delivery system. June 2021 (page 200, note 5). Accessed July 26, 2021. http://www.medpac.gov/docs/default-source/default-document-library/jun21_medpac_report_to_congress_sec.pdf?sfvrsn=0

