# Supplementary material for: Association of Private Equity Investment in US Nursing Homes With the Quality and Cost of Care for Long-Stay Residents
Source: JAMA Health Forum. 2021 Nov 19;2(11):e213817. doi: 10.1001/jamahealthforum.2021.3817 (PMC8796926; doi:10.1001/jamahealthforum.2021.3817)
Supplement: Supplement. — eAppendix. Identification of Private Equity Nursing Homes eFigure. Sample Selection eTable 1. Total Acquisitions by Private Equity Firms eTable 2. Chronic and Potentially Disabling Conditions eTable 3. Changes in Quality and Costs After Private Equity Acquisition Compared With For-Profit Nursing Homes Without Private Equity Ownership Using a Washout Period eTable 4. Changes in Quality and Costs After Private Equity Acquisition Compared With For-Profit Nursing Homes Without Private Equity Ownership Using a Balanced Nursing Home Panel eTable 5. Pre-Trend Tests to Examine Outcomes of Private Equity Nursing Homes and For-Profit Nursing Homes Without Private Equity Ownership Prior to Acquisition [file jamahealthforum-e213817-s001.pdf]

# Supplementary Online Content

Braun RT, Jung HY, Casalino LP, Myslinski Z, Unruh MA. Association of private equity investment in US nursing homes with the quality and cost of care for long-stay residents. *JAMA Health Forum.* 2021;2(11):e213817. doi:10.1001/jamahealthforum.2021.3817

**eAppendix.** Identification of Private Equity Nursing Homes
**eFigure.** Sample Selection
**eTable 1.** Total Acquisitions by Private Equity Firms
**eTable 2.** Chronic and Potentially Disabling Conditions
**eTable 3.** Changes in Quality and Costs After Private Equity Acquisition Compared With For-Profit Nursing Homes Without Private Equity Ownership Using a Washout Period
**eTable 4.** Changes in Quality and Costs After Private Equity Acquisition Compared With For-Profit Nursing Homes Without Private Equity Ownership Using a Balanced Nursing Home Panel
**eTable 5.** Pre-Trend Tests to Examine Outcomes of Private Equity Nursing Homes and For-Profit Nursing Homes Without Private Equity Ownership Prior to Acquisition

This supplementary material has been provided by the authors to give readers additional information about their work.

© 2021 Braun RT et al. *JAMA Health Forum.*

## **eAppendix.** Identification of Private Equity Nursing Homes

We identified nursing home acquisitions by private equity (PE) firms using a previously established methodology.[1] Acquisitions were identified from 2010 to 2020 using the S&P Capital IQ, Irving Levin Associates Health Care M&A, and Centers for Medicare & Medicaid Services (CMS) Nursing Home Compare Ownership databases, followed by web-based searches. These databases report transactions, including the acquisition announcement date, the name of the acquired nursing home, the platform nursing home that acquired the nursing home, and the PE firm that owns the nursing home. The Nursing Home Compare database provides a CMS Certification Number (CCN), nursing home name, address, owner name, and the date that ownership began.

We confirmed PE acquisitions in the S&P and Irving Levin databases by manually reviewing each acquirer's business profile using the CB Insights, Bloomberg Businessweek, Pitchbook, the acquirer's websites, and web-based searches to examine whether they were a PE firm or PE-backed platform nursing home. Next, we used key word searches in the Nursing home Compare database to identify PE firms and PE-backed platform nursing homes that were not in the S&P and Irving Levin databases. Identified acquisitions were then matched to the CMS Provider of Services file using nursing home name and address to obtain the CCN. More details on the list of PE firms that acquired nursing homes are provided in eTable 1.

**eReference.**

1. Braun RT, Yun H, Casalino LP, et al. Comparative Performance of Private Equity–Owned US Nursing Homes During the COVID-19 Pandemic. JAMA network open 2020;3:e2026702-e.

© 2021 Braun RT et al. *JAMA Health Forum.*

## **eFigure.** Sample Selection

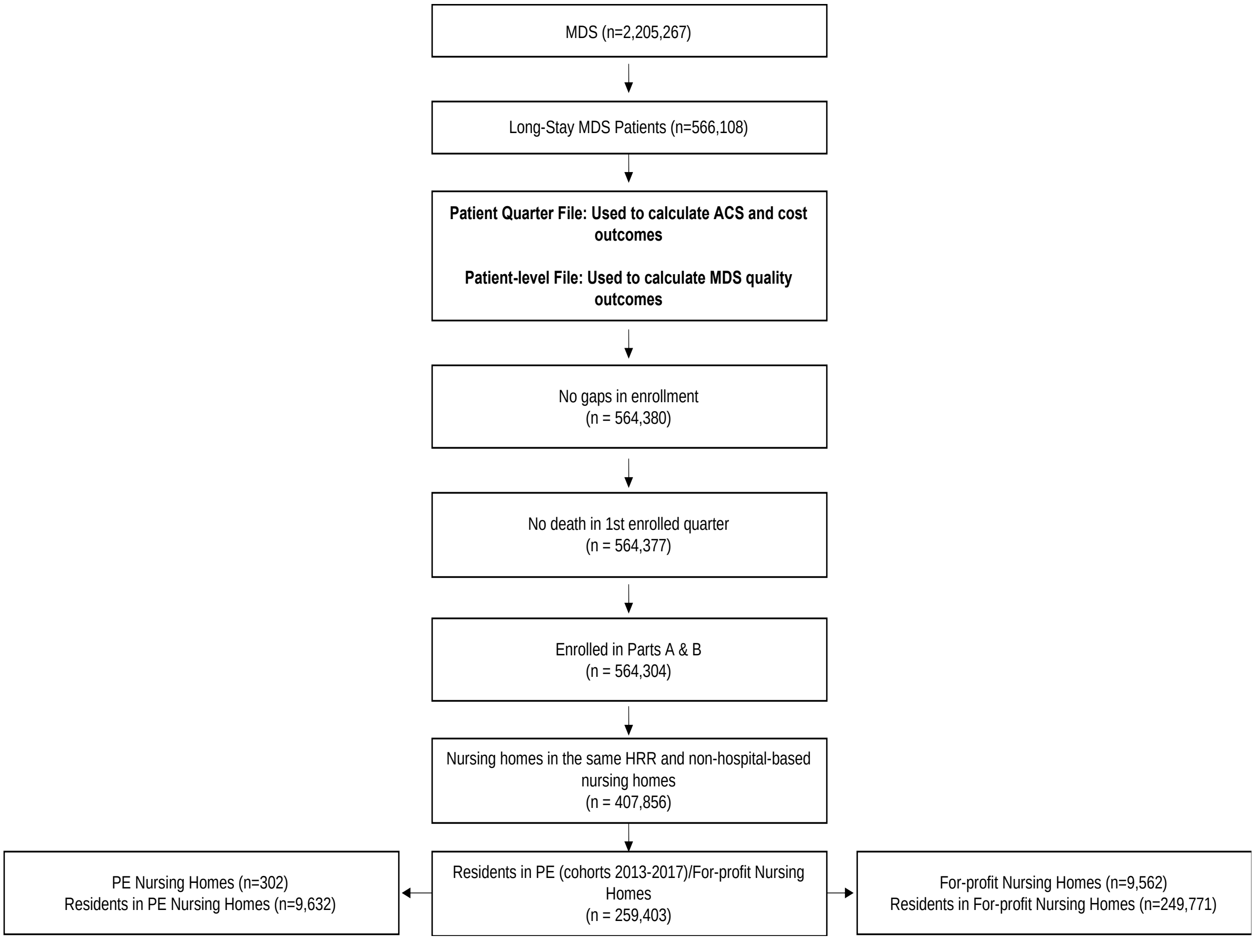


MDS: Minimum Data Set; ACS: ambulatory care sensitive; HRR: Hospital Referral Region; PE: private equity

© 2021 Braun RT et al. *JAMA Health Forum.*

## eTable 1. Total Acquisitions by Private Equity Firms

| PE Firms | Number of Transactions[1] | Number of Transactions in Cohorts (2013-2017)[1] |
|---|---|---|
| Arcapita | 1 | 0 |
| BayBridge Capital | 8 | 6 |
| Beechwood Capital | 1 | 0 |
| Birchwood Healthcare | 5 | 3 |
| Blue Ridge Capital | 1 | 0 |
| BlueMountain Capital | 2 | 1 |
| Cambridge Investment & Finance | 1 | 0 |
| Capital Senior Ventures | 1 | 1 |
| Capital Square 1031 | 1 | 0 |
| Cascade Partners | 8 | 3 |
| Centre Partners | 6 | 0 |
| Colonial Care | 1 | 0 |
| Colony Capital | 1 | 1 |
| Consulate Health Care | 1 | 0 |
| EEF Capital | 5 | 1 |
| Esplanade Capital | 1 | 0 |
| Falcon Capital | 2 | 2 |
| Focus Healthcare Partners | 1 | 0 |
| Formation Capital | 4 | 1 |
| Fortress Investment Group | 1 | 0 |
| Fulcrum Growth Partners | 2 | 2 |
| Genesis Healthcare | 49 | 0 |
| GI Partners | 1 | 0 |
| Golden Gate National Senior Care | 1 | 0 |
| Grubb & Ellis Equity Advisors | 1 | 0 |
| Harrison Street Real Estate Capital | 1 | 0 |
| HCR Manor Care | 5 | 0 |
| Investment360 | 2 | 2 |
| Javelin Capital Partners | 1 | 0 |
| Jeffersontown Opco | 1 | 0 |
| JER Partners | 2 | 0 |
| Kindred Healthcare | 2 | 0 |
| KKR & Co. | 1 | 0 |
| Liberty Group | 1 | 1 |
| Madison Partners | 2 | 2 |
| Millennium Management | 1 | 0 |
| Mission Healthcare | 7 | 2 |
| MVI Health Center | 1 | 0 |

© 2021 Braun RT et al. *JAMA Health Forum.*

| | | |
|---|---|---|
| National Senior Care | 4 | 2 |
| Nationwide Health Properties | 1 | 0 |
| O&M Investments | 6 | 2 |
| Onex | 8 | 7 |
| Pathfinder Partners | 1 | 1 |
| Peace Capital | 4 | 2 |
| Pearl Senior Care | 2 | 0 |
| Plum Healthcare Group | 1 | 0 |
| Pritok Capital | 5 | 5 |
| ROC Seniors Australia Investment Company | 1 | 1 |
| ROC Seniors Housing Holdings | 3 | 2 |
| RSF Partners | 1 | 1 |
| Signature Healthcare | 16 | 8 |
| Silverstone Health Care | 1 | 0 |
| Stockwell Capital | 1 | 0 |
| TD Capital | 1 | 0 |
| The Carlyle Group | 1 | 0 |
| The Portopiccolo Group | 1 | 0 |
| Tiptree Financial Partners | 1 | 0 |
| Trilogy Health Services | 9 | 3 |
| Tryko Partners | 9 | 5 |
| Vanguard Healthcare | 4 | 1 |
| Vista Healthcare Investments | 1 | 1 |
| Warburg Pincus | 1 | 0 |
| Windward Health Partners | 3 | 3 |
| *Unidentified Investor Groups* | 23 | 7 |
| **Total Transactions** | **242** | **79** |

[1] There were a total of 242 transactions merged on to claims data. Of the 242 transactions, 79 were between 2013-2017.

© 2021 Braun RT et al. *JAMA Health Forum.*

# eTable 2. Chronic and Potentially Disabling Conditions[1]

| | Chronic Condition |
|---|---|
| 1 | chronic_alzh_demen |
| 2 | chronic_alzh |
| 3 | chronic_ami |
| 4 | chronic_anemia |
| 5 | chronic_asthma |
| 6 | chronic_atrial_fib |
| 7 | chronic_cancer_breast |
| 8 | chronic_cancer_colorectal |
| 9 | chronic_cancer_endometrial |
| 10 | chronic_cancer_lung |
| 11 | chronic_cancer_prostate |
| 12 | chronic_cataract |
| 13 | chronic_chf |
| 14 | chronic_chronickidney |
| 15 | chronic_copd |
| 16 | chronic_depression |
| 17 | chronic_diabetes |
| 18 | chronic_glaucoma |
| 19 | chronic_hip_fracture |
| 20 | chronic_hyperl |
| 21 | chronic_hyperp |
| 22 | chronic_hypert |
| 23 | chronic_hypoth |
| 24 | chronic_ischemicheart |
| 25 | chronic_osteoporosis |
| 26 | chronic_ra_oa |
| 27 | chronic_stroke_tia |
| 28 | chronic_acp |
| 29 | chronic_alco |
| 30 | chronic_anxi |
| 31 | chronic_autism |
| 32 | chronic_bipl |
| 33 | chronic_brainj |
| 34 | chronic_cerpal |
| 35 | chronic_cysfib |
| 36 | chronic_depsn |
| 37 | chronic_drug |
| 38 | chronic_epilep |
| 39 | chronic_fibro |
| 40 | chronic_hearim |

© 2021 Braun RT et al. *JAMA Health Forum.*

| | |
|---|---|
| 41 | chronic_hepviral |
| 42 | chronic_hivaids |
| 43 | chronic_intdis |
| 44 | chronic_leadis |
| 45 | chronic_leuklymph |
| 46 | chronic_liver |
| 47 | chronic_migraine |
| 48 | chronic_mobimp |
| 49 | chronic_mulscl |
| 50 | chronic_musdys |
| 51 | chronic_obesity |
| 52 | chronic_othdel |
| 53 | chronic_oud_any |
| 54 | chronic_oud_dx |
| 55 | chronic_oud_hosp |
| 56 | chronic_oud_mat |
| 57 | chronic_psds |
| 58 | chronic_ptra |
| 59 | chronic_pvd |
| 60 | chronic_schi |
| 61 | chronic_schiot |
| 62 | chronic_spibif |
| 63 | chronic_spiinj |
| 64 | chronic_toba |
| 65 | chronic_ulcers |
| 66 | chronic_visual |

1. Centers for Medicare and Medicaid Services. Chronic Conditions Data Warehouse. (Accessed January 25, 2021, at https://www2.ccwdata.org/web/guest/condition-categories)

© 2021 Braun RT et al. *JAMA Health Forum.*

## eTable 3. Changes in Quality and Costs After Private Equity Acquisition Compared With For-Profit Nursing Homes Without Private Equity Ownership Using a Washout Period

| Outcome | Adjusted | P-Value |
| --- | --- | --- |
| *Quality Measures* | | |
| Emergency Department visit (binary, n = 2,369,600) | 2.0 [0.0-3.0] | .027 |
| Hospitalization (binary, n = 2,369,600) | 1.0 [0.0-1.0] | .021 |
| *Cost Measures* | | |
| Total costs (n=2,369,600) — $ | 217.53 [-77.60-512.66] | .15 |
| *Minimum Data Set Quality Measures* | | |
| Antipsychotic medications (binary, n = 229,520) | .00 [-1.0-2.0] | > 0.1 |
| Severe pain (binary, n = 216,302) | .00 [-1.0-2.0] | > 0.1 |
| Pressure ulcers (binary, n = 276,979) | 1.0 [-0.4-1.0] | > 0.1 |

Observations in the year of acquisition were considered the washout period and excluded from the analysis.

Linear regressions were used for estimation. All models included the following covariates: age (65-69, 70-74, 75-79, 80-84, 85+), race (white, black, other non-white race), sex, dual-eligibility for Medicare and Medicaid, indicators for 66 chronic and potentially disabling conditions used for risk adjustment (see eTable 2 for a list of the chronic conditions), activities of daily living (ADL) score at initial assessment (scale 1-28), and severe cognitive impairment (scores greater than 3 on the 4-point Cognitive Function Scale). Nursing home characteristics included occupancy rate, an indicator for multi-facility chain affiliation, total number of beds, terciles of the distributions of the percentage of patients covered by Medicare and the percentage covered by Medicaid. Other covariates included fixed-effects for quarter, year, nursing home, Hospital Referral Region (HRR), and HRR interacted with year.

Standard errors were adjusted for clustering at the level of the nursing home.

95% confidence intervals in brackets

[1] Relative changes were derived from the sample by dividing the adjusted estimates for all outcomes by the unadjusted mean of the outcomes in the pre-acquisition period (year 2012).

© 2021 Braun RT et al. *JAMA Health Forum.*

## eTable 4. Changes in Quality and Costs After Private Equity Acquisition Compared With For-Profit Nursing Homes Without Private Equity Ownership Using a Balanced Nursing Home Panel

| | **Preacquisition, 2012** | | **Differential Change** | | |
|---|---|---|---|---|---|
| **Outcome** | **Complete Sample** | | **Adjusted** | **P-Value** | **Relative Change**[1] |
| *Quality Measures* | | | | | |
| Emergency Department visit (binary, n = 2,235,339) | 15.0 | | 2.0 [0.3-3.3] | .019 | 13.3% |
| Hospitalization (binary, n = 2,235,339) | 11.0 | | 1.0 [0.3-1.0] | .002 | 9.1% |
| *Cost Measure* | | | | | |
| Total costs (n = 2,235,339) — $ | 6,945.90 | | 310.90 [76.82 – 545.10] | .009 | 4.5% |
| *Minimum Data Set Quality Measures* | | | | | |
| Antipsychotic medications (n = 182,982) | 22.0 | | 0.2 [-1.14-2.0] | > .1 | .9% |
| Severe pain (n = 171,186) | 9.0 | | 1.0 [-1.0-2.0] | > .1 | 11.1% |
| Pressure ulcers (n = 219,049) | 4.0 | | 1.0 [-.4 - 1.4] | > .1 | 25% |

When we restrict the sample to nursing homes that were present across all years of the study period, there were 275 private equity-owned nursing homes and 8,407 for-profit nursing homes never owned by private equity firms in the same Hospital Referral Regions.

Linear regressions were used for estimation. All models included the following covariates: age (65-69, 70-74, 75-79, 80-84, 85+), race (white, black, other non-white race), sex, dual-eligibility for Medicare and Medicaid, indicators for 66 chronic and potentially disabling conditions used for risk adjustment (see eTable 2 for a list of the chronic conditions), activities of daily living (ADL) score at initial assessment (scale 1-28), and severe cognitive impairment (scores greater than 3 on the 4-point Cognitive Function Scale). Nursing home characteristics included occupancy rate, an indicator for multi-facility chain affiliation, total number of beds, terciles of the distributions of the percentage of patients covered by Medicare and the percentage covered by Medicaid. Other covariates included fixed-effects for quarter, year, nursing home, Hospital Referral Region (HRR), and HRR interacted with year.

Standard errors were adjusted for clustering at the level of the nursing home.

95% confidence intervals in brackets

[1] Relative changes were derived from the sample by dividing the adjusted estimates for all outcomes by the unadjusted mean of the outcomes in the pre-acquisition period (year 2012).

© 2021 Braun RT et al. *JAMA Health Forum.*

# eTable 5. Pre-Trend Tests to Examine Outcomes of Private Equity Nursing Homes and For-Profit Nursing Homes Without Private Equity Ownership Prior to Acquisition

| | Emergency Department Visits (n = 2,383,491) | | Hospitalizations (n = 2,383,491) | | Total Cost of Care (n = 2,383,491) | |
|---|---|---|---|---|---|---|
| yminus5 | -3** | [-5, -1] | -1** | [-2, -0.3] | 136.4 | [-223.5, 496.4] |
| yminus4 | -2 | [-5, 2] | -1 | [-1, 0.2] | -272.0 | [-704.8, 160.9] |
| yminus3 | 0.2 | [-2, 3] | -1** | [-2, -0.4] | -383.9* | [-688.6, -79.10] |
| yminus2 | -0.2 | [-2, 2] | -0.1 | [-1, 0.4] | -50.24 | [-319.3, 218.8] |
| yminus1 | N/A | N/A | N/A | N/A | N/A | N/A |
| yplus0 | 1* | [0.3, 2] | 0.4 | [-0.4, 1] | 208.2 | [-70.78, 487.1] |
| yplus1 | 2*** | [1, 3] | 0.5 | [-0.3, 1] | 197.7 | [-125.8, 521.3] |
| yplus2 | 1 | [-1, 3] | 0.3 | [-1, 1] | 82.3 | [-328.9, 493.5] |
| yplus3 | 0.3 | [-2, 3] | -1 | [-2, 0.3] | 92.84 | [-458.6, 644.3] |
| yplus4 | 1 | [-2, 5] | 2* | [0.1, 3] | -24.79 | [-931.1, 881.5] |
| yplus5 | -6* | [-10, -0.2] | -1.0 | [-5, 3] | -1075 | [-3311.6, 1161.4] |

| | Antipsychotic Medication (n = 230,687) | | Severe Pain (n = 217,284) | | Pressure Ulcers (n = 278,188) | |
|---|---|---|---|---|---|---|
| yminus5 | 2 | [-2, 5] | -0.1 | [-5, 5] | 0.3 | [-1, 2] |
| yminus4 | 4 | [-1, 8] | -2 | [-5, 1] | -0.4 | [-2, 1] |
| yminus3 | -0.4 | [-3, 2] | -4*** | [-6, -2] | -0.4 | [-2, 1] |
| yminus2 | 1 | [-2, 5] | -2 | [-5, 1] | 0.03 | [-2, 2] |
| yminus1 | N/A | N/A | N/A | N/A | N/A | N/A |
| yplus0 | 0.4 | [-2, 3] | -2* | [-5, -0.1] | 0.3 | [-0.1, 1] |
| yplus1 | -0.1 | [-3, 3] | -1 | [-4, 2] | 1 | [-1, 2] |
| yplus2 | 3 | [-0.02, 6] | -1 | [-4, 2] | 0.4 | [-2, 2] |
| yplus3 | -0.1 | [-4, 3] | -1 | [-4, 2] | 0.4 | [-1, 2] |
| yplus4 | -2 | [-7, 4] | -2 | [-5, 2] | 1 | [-4, 5] |
| yplus5 | 16** | [4, 30] | 7 | [-6, 20] | -1 | [-10, 7] |

95% confidence intervals in brackets
* p<0.05, ** p<0.01, *** p<0.001

Linear regressions were used for estimation. All models included the following covariates: age (65-69, 70-74, 75-79, 80-84, 85+), race (white, black, other non-white race), sex, dual-eligibility for Medicare and Medicaid, indicators for 66 chronic and potentially disabling conditions used for risk adjustment (see eTable 2 for list of chronic conditions), activities of daily living (ADL) score at initial assessment (scale 1-28), and severe cognitive impairment (values greater than 3 on a 4-point Cognitive Function Scale). Nursing home characteristics included occupancy rate, an indicator for multi- facility chain affiliation, total number of beds, terciles of the distributions of the percentage of patients covered by Medicare and percentage covered by Medicaid. Other covariates included fixed-effects for quarter, year, nursing home, Hospital Referral Region (HRR), and HRR interacted with year.

Standard errors were adjusted for clustering at the level of the nursing home.

© 2021 Braun RT et al. *JAMA Health Forum.*
